# Neuropsychiatric Adverse Effects of Synthetic Glucocorticoids: A Systematic Review and Meta-Analysis

**DOI:** 10.1210/clinem/dgad701

**Published:** 2023-12-01

**Authors:** Anne-Sophie C A M Koning, Merel van der Meulen, Daphne Schaap, Djaina D Satoer, Christiaan H Vinkers, Elisabeth F C van Rossum, Wouter R van Furth, Alberto M Pereira, Onno C Meijer, Olaf M Dekkers

**Affiliations:** Department of Medicine, Division of Endocrinology, Leiden University Medical Center, Albinusdreef 2, 2333 ZA Leiden, The Netherlands; Department of Medicine, Division of Endocrinology, Leiden University Medical Center, Albinusdreef 2, 2333 ZA Leiden, The Netherlands; Department of Medicine, Division of Endocrinology, Leiden University Medical Center, Albinusdreef 2, 2333 ZA Leiden, The Netherlands; Department of Neurosurgery, Erasmus MC—University Medical Center Rotterdam, Dr. Molewaterplein 40, 3015 GD Rotterdam, The Netherlands; Department of Psychiatry and Department of Anatomy and Neurosciences, Amsterdam University Medical Center, Location VUMC, De Boelelaan 1117, 1081 HV Amsterdam, The Netherlands; Academic Working Place Depression, GGZ InGeest, Oldenaller 1, 1081 HJ Amsterdam, The Netherlands; Amsterdam Neuroscience (Mood, Anxiety, Psychosis, Stress & Sleep Program) and Amsterdam Public Health (Mental Health Program) Research Institutes, Amsterdam, The Netherlands; Department of Internal Medicine, Division of Endocrinology, Erasmus MC, University Medical Center Rotterdam, Dr. Molewaterplein 40, 3015 GD Rotterdam, The Netherlands; University Neurosurgical Center Holland, Leiden University Medical Center, Haaglanden Medical Center and Haga Teaching Hospitals, Leiden and The Hague, The Netherlands; Department of Endocrinology and Metabolism, Amsterdam University Medical Center, Location University of Amsterdam, Meibergdreef 9, 1105 AZ Amsterdam, The Netherlands; Amsterdam Gastroenterology Endocrinology Metabolism, Meibergdreef 9, 1105 AZ Amsterdam, The Netherlands; Department of Medicine, Division of Endocrinology, Leiden University Medical Center, Albinusdreef 2, 2333 ZA Leiden, The Netherlands; Department of Medicine, Division of Endocrinology, Leiden University Medical Center, Albinusdreef 2, 2333 ZA Leiden, The Netherlands

**Keywords:** neuropsychiatry, glucocorticoids, depression, anxiety, prednisone, dexamethasone

## Abstract

**Context:**

Synthetic glucocorticoids are widely used to treat patients with a broad range of diseases. While efficacious, glucocorticoids can be accompanied by neuropsychiatric adverse effects.

**Objective:**

This systematic review and meta-analysis assesses and quantifies the proportion of different neuropsychiatric adverse effects in patients using synthetic glucocorticoids.

**Methods:**

Six electronic databases were searched to identify potentially relevant studies. Randomized controlled trials, cohort studies, and cross-sectional studies assessing psychiatric side effects of glucocorticoids measured with validated questionnaires were eligible. Risk of bias was assessed with RoB 2, ROBINS-I, and AXIS appraisal tool. For proportions of neuropsychiatric outcomes, we pooled proportions, and when possible, differences in questionnaire scores between glucocorticoid users and nonusers were expressed as standardized mean differences (SMD). Data were pooled in a random-effects logistic regression model.

**Results:**

We included 49 studies with heterogeneity in study populations, type, dose, and duration of glucocorticoids. For glucocorticoid users, meta-analysis showed a proportion of 22% for depression (95% CI, 14%-33%), 11% for mania (2%-46%), 8% for anxiety (2%-25%), 16% for delirium (6%-36%), and 52% for behavioral changes (42%-61%). Questionnaire scores for depression (SMD of 0.80 [95% CI 0.35-1.26]), and mania (0.78 [0.14-1.42]) were higher than in controls, indicating more depressive and manic symptoms following glucocorticoid use.

**Conclusion:**

The heterogeneity of glucocorticoid use is reflected in the available studies. Despite this heterogeneity, the proportion of neuropsychiatric adverse effects in glucocorticoid users is high. The most substantial associations with glucocorticoid use were found for depression and mania. Upon starting glucocorticoid treatment, awareness of possible psychiatric side effects is essential. More structured studies on incidence and potential pathways of neuropsychiatric side effects of prescribed glucocorticoids are clearly needed.

Glucocorticoids are widely used drugs because of their anti-inflammatory effects, with an annual prevalence estimated at approximately 24% in 2022 in The Netherlands, including systemic, inhaled, nasal, and topical glucocorticoids (1). Although these drugs are very efficacious, they can be accompanied by (severe) side effects, including neuropsychiatric symptoms and disorders, such as mood changes, depression, anxiety, mania, delirium, and even suicidality, with devastating effects on quality of life (2, 3). While at low doses many synthetic glucocorticoids do not penetrate the blood-brain barrier (4), their use has been associated with a higher likelihood of mood and anxiety disorders (5). This includes particular types of inhaled glucocorticoids. In terms of biological effects, van der Meulen et al (2022) found associations of systemic and inhaled glucocorticoid use with reduced white matter integrity in the brain in a large sample from the UK Biobank (6). In addition, Kachroo et al (2022) found systemic effects in metabolomic profiles of inhaled glucocorticoids, even in use of low-dose glucocorticoids (7). Next to macroscopic and metabolomic changes, there is a plethora of neurochemical processes that may be affected by glucocorticoid medication (8-10).

The endogenous glucocorticoid hormones cortisol and corticosterone target 2 receptor types, the high affinity mineralocorticoid receptor (MR) and the lower affinity glucocorticoid receptor (GR). In contrast, synthetic glucocorticoids have been developed based on a high affinity for GR, which mediates their many and potent anti-inflammatory effects even though relative affinities differ (11). The most common assumption is that overactivation of the GR—in particular during the natural trough of cortisol secretion (12)—mediates the adverse effects of glucocorticoid medication on the brain. A complementary view is that suppressed cortisol secretion via GR-dependent negative feedback contributes to mental side effects by depleting the MR from its ligand. This notion leads to the prediction that re-activation of the MR with cortisol add-on will counteract these adverse effects (13-15). There is preliminary evidence that this approach is beneficial in patients with leukemia (16), but this finding is not replicated in the same study population that included only patients with clinically relevant neurobehavioral problems (17, 18). Currently, a randomized controlled trial (RCT) is ongoing to investigate the prevention of neuropsychiatric adverse effects caused by dexamethasone in an adult brain tumor population (19). The occurrence of central side effects is expected to depend on the potency to activate MR and GR, pharmacokinetic characteristics route of administration, dose, and duration of exposure to the particular drug. It is not exactly known in what frequency psychiatric adverse effects occur with the different glucocorticoids that are available.

Recently, a systematic review identified adverse effects of systemic glucocorticoid therapy (20). The study distinguished 4 categories for glucocorticoid side effects: physical symptoms, psychological symptoms, effects on participation (eg, impact on work and impact on family role), and contextual factors (eg, support of family and friends). Physical and psychological symptoms were most prominent, including irritability and mood swings. We aimed to expand this work by focusing specifically on neuropsychiatric adverse effects of glucocorticoids measured with validated questionnaires and by calculating proportions. Furthermore, we aimed to separate the effects of the different types of glucocorticoids.

The aim of this review is therefore to describe neuropsychiatric adverse effects in glucocorticoid users and to estimate the proportion of patients developing neuropsychiatric adverse effects. Secondly, when possible, these adverse effects were quantified by investigating the differences in neuropsychiatric questionnaire scores between glucocorticoid users and nonusers.

## Methods

This systematic review and meta-analysis are reported according to the Preferred Reporting Items for Systematic Review and Meta-Analyses (PRISMA) statement (21). The study was registered in the PROSPERO international prospective register of systematic reviews with registration number CRD42022285282.

### Search Strategy

A literature search was conducted in September 2021, and updated in May 2023, to identify studies describing psychiatric effects of glucocorticoids. The following databases were systematically searched for relevant studies: PubMed, Embase, Web of Science, Cochrane Library, PsycINFO, and Academic Search Premier. Data were processed to an EndNote X9 database (Clarivate Analytics, Philadelphia, PA, US). The complete search strategy is presented in Supplement 1 (22).

### Eligibility Criteria and Article Selection

Studies that were RCTs, cohort, and cross-sectional studies assessing psychiatric side effects during or right after any type of glucocorticoids were eligible for inclusion; studies in patients using glucocorticoids as endocrine substitution were not considered. Studies were considered irrespective of the indication for glucocorticoid use. Studies were excluded if glucocorticoids were used to treat psychiatric effects, and if psychiatric effects were not measured with validated questionnaires. Non-English papers and congress abstracts were not considered. Studies assessing questionnaires a month after glucocorticoid use were not considered.

Studies were screened by title and abstract and potentially relevant studies were reviewed by full-text analysis. Screening of studies, data extraction, and risk of bias assessment were performed by 2 independent reviewers (A-S.K. and D.S.). Disagreement was solved through discussion. If discussion failed to reach consensus a third reviewer was consulted (M.M.).

### Data Extraction

The following data were extracted, if available: study population, mean age, percentage female subjects, type and dosage of glucocorticoid, duration of glucocorticoid use, psychiatric outcome measures, and follow-up duration (cohort studies). The complete table with extracted data is presented in Supplemental Table S1 (22). In studies with additional medication in combination with glucocorticoids, the data from the glucocorticoids-only group (and placebo) were extracted.

In studies that used more than one questionnaire for the same symptom, the questionnaire that was used most frequently across all included studies was chosen for analysis. Furthermore, only 3 studies investigated prednisolone, and because this drug is the biologically active form of prednisone, we decided to combine the prednisolone with the prednisone studies.

### Definition of Neuropsychiatric Adverse Effects

Studies using validated questionnaires were eligible for inclusion. Questionnaire-specific cutoffs were used to determine the presence or absence of the different neuropsychiatric conditions.

### Risk of Bias Assessment

To assess the risk of bias, the revised Cochrane risk of bias tool was used for randomized trials (RoB 2), and the risk of bias in nonrandomized studies of interventions (ROBINS-I) assessment tool was used for cohort studies (23, 24). Studies were defined as having a low, moderate, or high risk of bias. For cross-sectional studies the AXIS appraisal tool was used, which does not have a summarizing qualifying score (25). The scoring system is presented in Supplementary Table S2 (22).

### Study Endpoints

The primary study outcome was the pooled proportion of different neuropsychiatric adverse effects in glucocorticoid users measured with validated questionnaires or classified according to the DSM-IV or ICD-9/10. Secondly, differences in neuropsychiatric questionnaire scores between glucocorticoid users and nonusers were investigated.

### Statistical Analysis

The main outcomes were the pooled proportion of patients with neuropsychiatric adverse effects. A random-effects logistic regression model was performed to pool proportions when there were 4 or more studies for a specific analysis; proportions were reported as percentages including their 95% CI. Secondly, differences in neuropsychiatric questionnaire scores between glucocorticoid users and nonusers were investigated and pooled as standardized mean differences (SMD) to standardize different questionnaires. For the interpretation of SMD, an effect size of 0.2 indicates a small effect, 0.5 a moderate effect and 0.8 a large effect (26). When the SMD was not reported, pooled odds ratios (OR) were used. Heterogeneity between studies was presented with *I^2^* statistics. An *I^2^* of less than 25% is usually regarded as low heterogeneity, between 25% and 50% as moderate, and over 50% as high heterogeneity (27). Subgroup analyses were used to explore potential heterogeneity. All meta-analyses were visualized using forest plots. The “tidyverse” (version 2.0.0) (28) and “meta” (version 6.5-0) (29) packages for RStudio statistical software were used (R version 4.2.0; R Foundation for Statistical Computing, Vienna, Austria, 2016; https://www.R-project.org/).

## Results

### Study Selection

**Figure 1.**
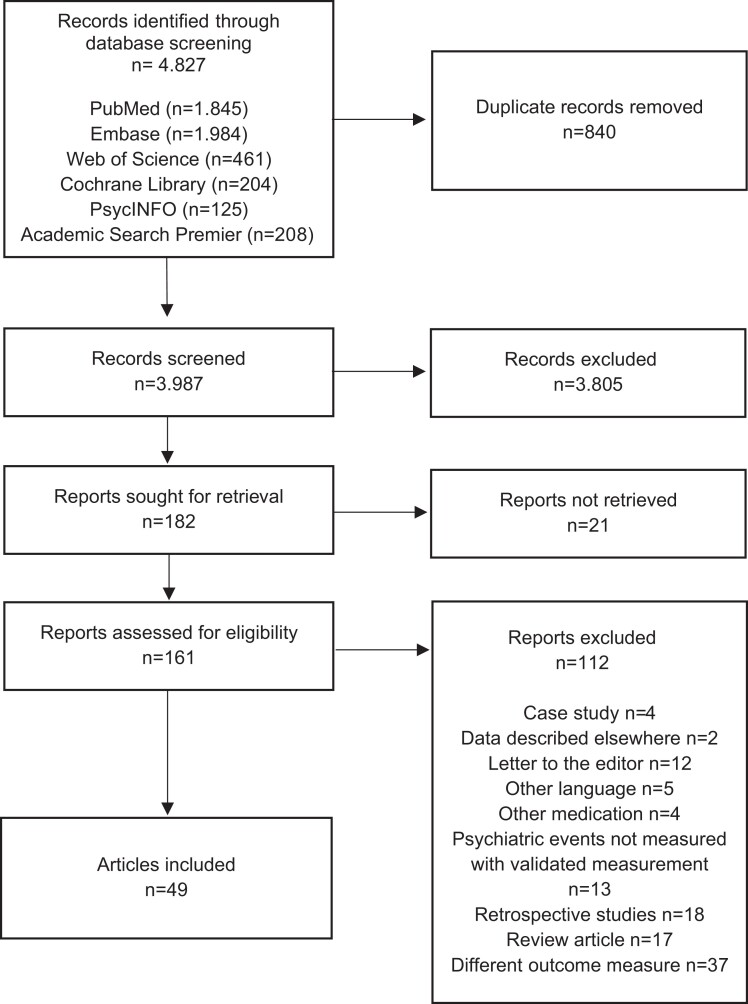
Flowchart of article screening and inclusion.

The literature search yielded 3987 unique studies. After exclusion of studies based on title and abstract, 197 full-text articles were screened. Finally, 49 studies were included (Fig. 1): 6 RCTs, 12 cross-sectional studies, and 31 cohort studies. A reference list of included studies is provided in Supplement 2 (22). Most studies were performed in the United States (n = 15), Europe (n = 12), and Canada (n = 9) and were published between 1981 and 2023; 23 (47%) were published after 2010.

### Risk of Bias Analysis

Of the 6 RCTs, 1 displayed a high risk, 4 displayed moderate risk, and 1 displayed low risk of bias. Of the 31 cohort studies, 25 showed high risk, and 6 presented with moderate risk of bias. The AXIS tool for cross-sectional studies did not provide a total score, but all 12 studies scored positively on recruiting a representative sample, scored positively on the appropriateness of the measurements being used, described the methods and basic data adequately, and scored positively on the results presented and justification in discussion and conclusions, which we interpreted as reasonable quality. The full risk of bias assessment is presented in Supplementary Table S2 (22).

### Study Characteristics

Characteristics of the 49 included studies are shown in Supplementary Table S1 (22). Of these, 39 studies were performed in adults, including healthy participants/general population (n = 5), and patients with different conditions (lung diseases (n = 8), combination of lung, rheumatic and internal ward patients (n = 4), diabetes mellitus (n = 1), systemic lupus erythematosus (n = 4), multiple sclerosis (n = 3), gastrointestinal patients (n = 1), neuromuscular disease (n = 1), plastic surgery (n = 1), hip fractures (n = 1), (critical) medical inpatients (n = 3), frozen shoulder (n = 1), cancer (n = 4), inflammatory bowel disease (n = 1), and neurological diseases (n = 1). Ten studies were performed in children, most of whom suffered from acute lymphoblastic leukemia (n = 6). Other diseases included nephrotic syndrome (n = 3) and asthma (n = 1).

#### Glucocorticoids: type, route of administration, duration, and dose

Prednisone (including prednisolone) was most frequently investigated (n = 15), followed by dexamethasone (n = 5) and methylprednisolone (n = 4). Eight studies did not report the type of glucocorticoid that was investigated, and 17 studies reported on a combination of different glucocorticoids.

In most studies, the route of administration was not reported (n = 21). Ten studies reported the use of oral glucocorticoids, 6 studies investigated intravenously administered glucocorticoids, only 1 study examined inhaled glucocorticoids, and 11 studies reported a combination of different administered glucocorticoids (eg, inhaled and systemic; or systemic, inhaled, topical, and nasal; or topical, inhaled, and oral).

Duration of glucocorticoid usage was highly heterogenous, ranging from one single administration to daily use for a few days or several months. To distinguish shorter (few days) from longer usage (weeks), we divided the duration into ≤8 days and >8 days. This distinction was based on initial data review and the goal to have a balance in the number of studies in the groups. Fourteen studies reported a duration of 8 or fewer days (median of 4 days; range, 1-8), and 20 studies for more than 8 days (median of 35 days; range, 11-4470), while 15 studies did not report the duration.

The dosage also differed considerably between studies. Studies reported cumulative daily dose, cumulative total dose, mean daily dose, and also mg/kg/day or only 1 single administration of glucocorticoid. Supplementary Table S1 contains dosages information per study (22).

#### Measured neuropsychiatric adverse effects

Neuropsychiatric adverse effects were measured with 39 different questionnaires (Supplementary Table S3) (22). Most commonly assessed were depressive symptoms (n = 24), mania (n = 13), behavior (n = 9), anxiety (n = 7), and delirium (n = 8). Seventeen studies assessed the presence of multiple neuropsychiatric adverse effects. Mood was assessed in 3 studies, 1 study measured obsessive compulsive disorder, and 6 medicine- or disease-specific questionnaires were used. In Supplementary Table S3, the different questionnaires are presented and arranged by the adverse effect or symptoms measured (22).

### Depression

#### Study characteristics

Twenty-four studies used depression-specific questionnaires to measure the presence of depressive symptoms, most commonly with the Beck Depression Inventory (BDI) (10 studies) and the Hamilton Rating Scale for Depression (HRSD/HAM-D) (6 studies, of which 4 were from the same first author). Depression was also measured with other questionnaires: 4 disease- or medicine-specific questionnaires, and 5 multiple neuropsychiatric adverse effects questionnaires. Since some studies used more than 1 questionnaire, in total 28 studies used questionnaire for the measurement of depression.

#### Risk of bias

Of the 28 studies, 3 were RCTs. One RCT had low risk of bias, 1 had moderate risk, and 1 had high risk of bias due to missing outcome data. Sixteen studies were cohort studies, of which 3 had a moderate risk of bias and 13 had a high risk of bias. Nine studies were cross-sectional with reasonable quality.

#### Meta-analyses on the proportion of depression

**Figure 2.**
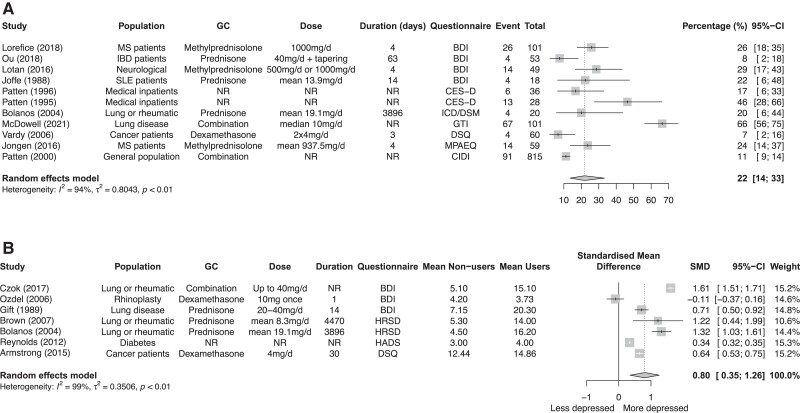
Meta-analysis on the percentage of depression in glucocorticoid users (A). Meta-analysis comparing depression scores between glucocorticoid users and nonusers (B).

Of the 28 studies, 12 articles contributed data for quantitative analyses. However, 1 study was excluded from analysis, because the presented prevalence data was that of the entire sample, and not as they mentioned, the glucocorticoid user group. We performed a meta-analysis of 11 studies that included a total of 1340 patients. The 11 study populations had a mean age ranging from 37.0 to 60.0 years. The pooled proportion of patients experiencing depressive symptoms with glucocorticoid usage was 22% (95% CI, 14%-33%; *I^2^* statistics = 94%) (Fig. 2A). Subgroup analyses for type of glucocorticoid and duration were performed and did not reduce heterogeneity (Supplementary Figure 1a and 1b) (22).

#### Meta-analysis comparing depression scores between glucocorticoid users and nonusers

Seven studies compared glucocorticoid users to nonusers. Higher scores on the depression questionnaires were found in glucocorticoid users compared to nonusers, SMD 0.80 (95% CI, 0.35-1.26) (Fig. 2B); this is regarded as a large effect (26). Subgroup analyses for type of glucocorticoid and duration are shown in Supplementary Figure 1c and 1d (22).

### Mania

#### Study characteristics

Thirteen studies measured symptoms of mania with questionnaires specific for mania, most commonly with the Young Mania Rating Scale (YMRS) (6 studies) and the Activation subscale of the Internal State Scale (AS-ISS) (5 studies). Mania was also measured with 1 disease- or medicine-specific questionnaire, 1 multiple neuropsychiatric adverse effects questionnaire, 2 with the Mood Disorder Questionnaire (MDQ), and 1 with the Present State Examination (PSE). Since some studies used more than 1 questionnaire, in total 14 studies mentioned the use of a questionnaire for the measurement of mania.

#### Risk of bias

The 14 studies included 2 RCTs. One RCT had moderate risk of bias and the other high risk, due to the score on missing outcome data. Nine studies were cohort studies, of which 4 had a moderate risk of bias, and 5 had a high risk of bias. Three studies were cross-sectional with reasonable quality.

#### Meta-analyses on the proportion of mania

**Figure 3.**
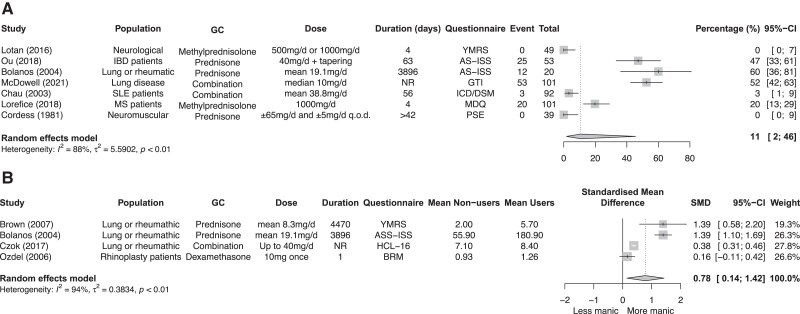
Meta-analysis on the percentage of mania in glucocorticoid users (A). Meta-analysis comparing mania scores between glucocorticoid users and nonusers (B).

Of the 14 studies, 7 articles contributed data for a meta-analysis that included a total of 455 patients, with a mean age ranging from 34.3 to 54.4 years. The pooled proportion of mania was 11% (95% CI, 2%-46%; *I^2^* 88%) (Fig. 3A). Subgroup analyses for type of glucocorticoid and duration were performed and are shown in the Supplementary Figures 2a and 2b (22).

#### Meta-analysis comparing mania scores between glucocorticoid users and nonusers

Four of the 14 studies compared glucocorticoid users to nonusers. Higher scores on the mania questionnaires were found in glucocorticoid users compared to nonusers: SMD 0.78 (95% CI, 0.14-1.42) (Fig. 3B); this is regarded as a large effect (26).

### Anxiety

#### Study characteristics

Seven studies used anxiety-specific questionnaires to measure the presence of anxiety symptoms, most commonly with the Hospital Anxiety and Depression Scale (HADS) (3 studies), and the Spielberger State Anxiety Index (2 studies). Anxiety was also measured with 1 disease- or medicine-specific questionnaire and 2 multiple neuropsychiatric adverse effects questionnaires. In total, 10 studies mentioned the use of a questionnaire for the measurement of anxiety.

#### Risk of bias

Of the 10 studies, 1 was an RCT with low risk of bias. Five studies were cohort studies, all with high risk of bias. Four studies were cross-sectional studies with reasonable quality.

#### Meta-analyses on the proportion of anxiety

**Figure 4.**
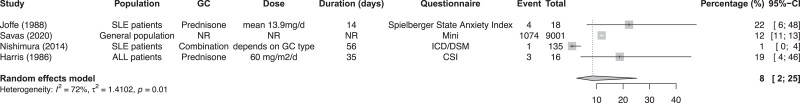
Meta-analysis on the percentage of anxiety in glucocorticoid users.

Four of the 10 studies contributed data for a meta-analysis, including a total of 9170 patients. The mean age ranged from 36.7 to 45.2 years (1 study only reported the range: 4 to 16). The pooled proportion of patients experiencing anxiety with glucocorticoid use was 8% (95% CI, 2%–25%; *I^2^* 72%) (Fig. 4). Since there was only 1 article that contributed data on glucocorticoid users compared to nonusers, no meta-analyses of the difference in anxiety questionnaire scores could be performed.

### Delirium

#### Study characteristics

Eight studies measured the presence of delirium symptoms with delirium-specific questionnaires, most commonly with the Confusion Assessment Method (CAM) (4 studies). Two studies used 2 questionnaires for the presence of delirium, and the presence of delirium was also measured with a multiple neuropsychiatric adverse effects questionnaire. Because some studies used more than 1 questionnaire, in total 7 studies mentioned the use of a questionnaire for the measurement of delirium.

#### Risk of bias

There were 2 RCTs that investigated delirium, and both RCTs had moderate risk of bias. Five were cohort studies, of which 1 had moderate risk of bias and the others had serious risk of bias.

#### Meta-analyses on the proportion of delirium

**Figure 5.**
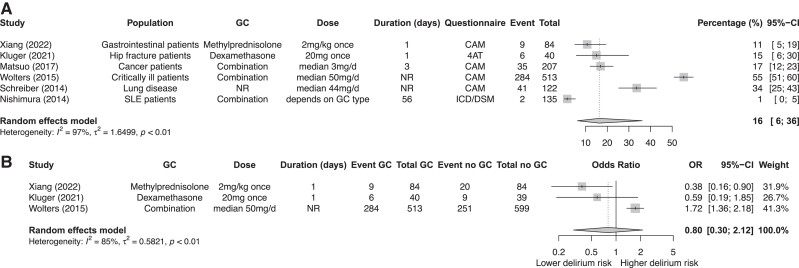
Meta-analysis on the percentage of delirium in glucocorticoid users (A). Meta-analysis comparing delirium scores between glucocorticoid users and nonusers (B).

Of the 7 studies, 6 contributed data for a meta-analysis that included a total of 1101 patients. The mean age ranged from 51.0 to 81.4 years (1 study did not report age). The pooled proportion of delirium in people with glucocorticoid use was 16% (95% CI, 6%-36%; *I^2^* 97%) (Fig. 5A). Subgroup analyses for type of glucocorticoid and duration were performed and are shown in the Supplementary Figure 3a and 3b (22).

#### Meta-analysis comparing delirium scores between glucocorticoid users and nonusers

Three studies contributed data that compared glucocorticoid users to nonusers. No clear association between glucocorticoid use and risk of delirium was found: OR 0.80 (95% CI, 0.30-2.12). This would suggest that glucocorticoid users have a lower risk for developing delirium. However, 2 RCTs were included and in both studies glucocorticoids were only administered once (Fig. 5B).

### Behavior

#### Study characteristics

Nine studies measured behavioral changes. Behavioral changes in relation to glucocorticoids are often investigated in children. Behavior is therefore only analyzed in children. The most commonly used questionnaire to assess behavior was the Child Behavior Checklist (CBCL) (6 studies). This questionnaire quantifies problematic behavior and skills in children, for example, anxious/depressed, aggression, social and attention problems. One study used 2 questionnaires, which results in a total of 8 studies assessing behavior.

#### Risk of bias

All studies, except one, were cohort studies, all with high risk of bias. One RCT was included with moderate risk of bias.

#### Meta-analyses on the proportion of behavior

**Figure 6.**
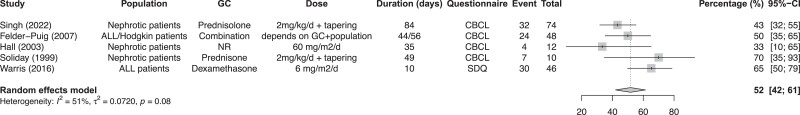
Meta-analysis on the percentage of behavioral changes in glucocorticoid users.

Five of the 8 studies contributed data for a meta-analysis, including a total of 190 patients. The mean age ranged from 4.0 to 14.3 years (1 study did not report age). The pooled proportion of children with behavioral changes with glucocorticoid use was 52% (95% CI, 42%-61%; *I^2^* 51%) (Fig. 6). Subgroup analyses for type of glucocorticoid and duration were performed and shown in Supplementary Figures 4a and 4b (22). No meta-analyses on the differences in behavioral changes between glucocorticoid users and controls could be performed since the studies were too heterogeneous in their endpoints.

## Discussion

We performed a systematic review and meta-analysis on the proportions of neuropsychiatric adverse effects of synthetic glucocorticoids. The pooled proportion could be investigated for several neuropsychiatric adverse effects, which varied from 22% for depression, 11% for mania, 8% for anxiety, and 16% for delirium. For both depression and mania, worse questionnaire scores were found in glucocorticoid users compared with nonusers. Importantly, the given percentages do not say anything about the causality of neuropsychiatric adverse effects. The numerical approach in the paper is two-fold. When comparing users with nonusers, the data show that glucocorticoid users have more neuropsychiatric symptoms, for example depressive symptoms (expressed as SMD). Although a single percentage in users does in no way mean that the full percentage is due to glucocorticoids, it shows that, for example, depression is rather common. The combination of these 2 numbers tells the clinicians that neuropsychiatric symptoms are common in glucocorticoid users, and may partly be related to glucocorticoid effects. It should be underlined that this is not an argument for stopping glucocorticoid treatment in these patients. Unfortunately, for some conditions (anxiety, behavioral changes), no comparative data were found.

Neuropsychiatric symptoms are well-known possible adverse effects of synthetic glucocorticoid usage, and are well-described in literature reviews, and studies investigating large databases (2, 3). However, a quantified risk for patients during glucocorticoid usage is largely unknown. A pooled analysis of adverse events of (only low to medium dose) glucocorticoids had previously been performed but only analyzed in patients with inflammatory diseases, and most included studies did not systematically assess adverse events (30). The latter will lead to lower reliability of reported rates of the various adverse events. As suggested by others, the use of patient reported outcome measures (PROs) may overcome this problem (20, 31). Depressive symptoms were mostly assessed with the Beck Depression Inventory, and Hamilton Rating Scale for Depression (HRSD/HAM-D). Mania was mostly assessed with the Young Mania Rating Scale (YMRS), and Activation subscale of the Internal State Scale (AS-ISS). For anxiety, the Hospital Anxiety and Depression Scale (HADS), and Spielberger State Anxiety Index were mostly used. Delirium was assessed most with the Confusion Assessment Method (CAM), and to measure behavioral changes in children, the Child Behavior Checklist (CBCL) was most used. Our study shows the need for a general assessment tool to capture neuropsychiatric glucocorticoid-related adverse effects, since we demonstrated the use of 39 different questionnaires in the 49 included studies.

The current study aimed to create an overview of proportions of neuropsychiatric adverse effects measured with validated questionnaires in any study population. Neuropsychiatric effects which were only registered as adverse events were not included, because it was unknown how systemically they had been registered. Our findings are therefore most likely underestimations of the true proportions. We showed pooled proportions of several neuropsychiatric symptoms, ranging from 8% to 22%. Another recent systemic review published by Cheah et al (2020) demonstrated that, next to weight gain and problems related to sleep, irritability and mood swings were the most common effects associated with the use of glucocorticoids, with a frequency effect size of 74%. The frequency effect sizes of depression or low mood was 43%, anxiety 39%, hyperactivity/euphoria 30%, and neuropsychiatric symptoms 9% (20). These percentages are higher compared with our findings. These authors used a different method, taking the number of studies containing a specific finding and dividing this number by the total number of studies, expressing it as a percentage, while the current study used proportion data from included studies. Our findings are fairly consistent with a study by Fardet et al (2007), who performed a cohort study in patients receiving prednisone (mean of 42 mg/day) for 3 months (32). They demonstrated that patients developed mood-related conditions, including irritability (25%), anxiety or depression (11.3%), manic episodes (3.8%), and euphoric hyperactivity (12.5%) (32). This study was not included in our analysis since it was not clear whether a validated questionnaire was used.

Pooled analysis comparing questionnaire scores between glucocorticoid users and nonusers could be performed for depression, mania, and delirium, although the analysis for delirium only included 3 studies. For depression and mania, a difference in questionnaire scores was found, with glucocorticoid users having higher or worse depression or mania scores compared to nonusers. The fact that depression and mania scores are worse following glucocorticoid use indicates that depressive and manic symptoms may need to be monitored during glucocorticoid treatment. For delirium, the pooled odds ratio showed a nonsignificant lower risk for delirium in glucocorticoid users, which could be explained by the duration of glucocorticoid use (for 2 of the 3 studies use was only a single administration); also, the confidence interval was wide, pointing toward uncertainty in the estimation. Delirium is a well-known adverse effect of glucocorticoids (3, 33, 34), but a single administration might not be enough to trigger this adverse effect. It should also be considered that if glucocorticoids indeed improve the underlying condition, this in itself can have a positive impact on neurocognitive symptoms.

Of the 49 included studies, less than half were included for quantitative analyses. Reporting of data was highly variable, and many studies did not compare users to nonusers. Furthermore, most studies were cohort studies and the majority had a serious risk of bias. It is important to note that there was substantial heterogeneity between the studies. The study population, type of glucocorticoid, the dose and duration were different between studies, which limits comparability. Additionally, these could all be confounding factors for the association between glucocorticoids and neuropsychiatry, especially in cohort studies. Data from RCTs are therefore preferred, but the present review only included 7 RCTs and these all differed in study population, type, dose, and duration of glucocorticoid, and used different outcomes, which made comparison or pooling of data not possible. The study population or the underlying disease can also have an effect on the psyche, such as in systemic lupus erythematosus (35, 36). Furthermore, higher dosage, long-term treatment, older age, and a history of a neuropsychiatric disorder are all known to be associated with a greater risk of glucocorticoid-induced neuropsychiatric symptoms (2). The questionnaire-specific cutoffs can contribute to the heterogeneity. In addition, many questionnaires often measure the presence or severity of symptoms, but do not actually diagnose patients with symptoms.

Furthermore, due to lack of power of subgroup analyses and multilayered heterogeneity, we were unable to identify differences in neuropsychiatric adverse effects for the different types of glucocorticoids. Still, it would be very interesting to investigate possible differences in neuropsychiatric symptoms between the various glucocorticoids, especially with the MR refill hypothesis in mind. This hypothesis states that underactivation of the MR due to synthetic glucocorticoid treatment may play a role in the development of neuropsychiatric side effects. Additional cortisol can bind to the empty MRs and receptor occupancy and/or balance will be restored (13). The potency of the synthetic glucocorticoids could be a factor of influence, in which higher GR potency might lead to a more rapid suppression of the hypothalamic-pituitary-adrenal axis. Moreover, a longer half-life might extend the effects, and the administration route could all influence the effects. Compared with endogenous cortisol, dexamethasone, prednisone, and prednisolone all have lower potency at the MR. The MR refill hypothesis makes it tempting to speculate that glucocorticoids with highest MR affinity may have the least psychiatric adverse effects. Of course, this should be investigated, and future studies could focus on the different adverse effects between the types of glucocorticoids, and further substantiate the MR refill hypothesis. In addition, it is theoretically possible that—for example, based on affinity and residence time at the receptor—specific GR ligands would have differential effects on target gene expression, but this has not been studied (37).

The MR refill hypothesis has already been investigated in different studies, including 3 RCTs. Two RCTs have been performed in pediatric patients with acute lymphoblastic leukemia. These patients were treated with dexamethasone (3 times 2 mg/m^2^ each day for 5 days) as part of their treatment. They were also treated with hydrocortisone or placebo (5, 3, and 2 mg/m^2^ each day) in a cross-sectional design. The first RCT demonstrated a positive effect of cortisol addition in a subgroup of children who experienced severe adverse effects; however, the second RCT could not replicate this finding (16, 17). The third study is an RCT in patients with a brain tumor who undergo surgery and receive high-dose dexamethasone (mostly 2 times 8 mg each day, or a minimal dose of at least 24 mg in 6 days) (19). This study is still ongoing and will give insights into the effects of add-on cortisol in an adult population.

In addition to the MR refill hypothesis, it is also conceivable that the adverse effects are mediated by the GR. In endogenous Cushing syndrome, psychiatric manifestations are an important part of the disease (38, 39). In this situation endogenous cortisol is highly elevated and occupies both the GR and MR (8). Importantly, genetic variation in the GR gene has also been shown to be associated with differences in GR sensitivity. GR polymorphisms associated with increased GR sensitivity have been shown to be related to increased physical and mental effects of glucocorticoids, and less pronounced adverse effects in corticosteroid users harboring GR resistant polymorphisms (40, 41). Therefore, neuropsychiatric sequelae could also be related to GR activity, and this would imply that glucocorticoids with highest GR affinity may be causal for the neuropsychiatric adverse effects.

Next to identifying differences in neuropsychiatric symptoms for the different types of glucocorticoids, it is of interest to study this in relation to the route of administration. Our review was not able to investigate this matter; however, a large UK Biobank study demonstrated that the use of both systemic and inhaled glucocorticoids is associated with reduced white matter integrity in the brain, which may contribute to the neuropsychiatric symptoms (6). Another recent study also demonstrated systemic effects in metabolomic profiles of inhaled glucocorticoids (7). In the current review, only one study examined inhaled glucocorticoids only, while in other studies the combination of inhaled with systemic, topical, or nasal glucocorticoids were investigated. Inhaled, topical, and nasal administration of glucocorticoids can induce adrenal insufficiency (42), indicating some degree of systemic action that is causing the negative feedback. Future research could focus on examining neuropsychiatric symptoms between the different administration routes.

Moreover, further research into the underlying mechanism of neuropsychiatric adverse effects is warranted, especially since the reasons why some individuals experience symptoms and some remain asymptomatic are unclear. Of interest are studies investigating GR genetics, in which, for example, a certain MR haplotype shows increased expression with associated positive effects (43-45). In addition, polymorphisms of the GR gene have also shown to be associated with the severity of (side) effects, in particular also with respect to inhaled corticosteroids, and may be taken into account in further studies (40, 41). In this context, it is of interest to mention that there is ongoing research into selective GR modulators that have the potential to enable targeted medication usage, achieving the desired effect without the accompanying adverse effects (46, 47).

There are some limitations that need to be discussed. First, there is the heterogeneity regarding study population, the underlying disease, type of glucocorticoid, the dose, and duration. Also, reporting of data was different between studies, for example the dosages were given in mg/d, mg/kg/d, and mean mg/kg (see Supplementary Table S1) (22). This makes a formal comparison difficult and resulted in small subgroups with low power for such subgroup analyses. Second, only a few studies could be included in the analysis comparing glucocorticoid users with nonusers, and residual confounding may be present thereby hampering a firm statement regarding causality. Lastly, it is important to mention that the current meta-analysis does not prove causality between glucocorticoid use and the development of neuropsychiatric adverse effects. However, we would like to stress that the discussion on causality should consider the full body of evidence, including preclinical data.

In order to get clear incidences of specific neuropsychiatric adverse effects, future studies should state in a detailed manner the type, dose, duration, and route of administration of the glucocorticoid used. We also showed the need for comparative evidence for some of the neuropsychiatric conditions (eg, anxiety). Furthermore, the use and study of standard dosages and durations would make it easier to investigate the effect of dose and duration on complaints. Also, the use of control groups, like cross-over studies, to compare psychiatric complaints between users and nonusers or off-on periods, and a general assessment tool to capture these complaints are preferred. This might give better insights in which synthetic glucocorticoids are the most likely culprits, including at what dose and/or duration. Moreover, studies are warranted to assess whether tapering doses, if the underlying disease allows, would relieve some of the neuropsychiatric symptoms.

In conclusion, this study demonstrates that the proportion of neuropsychiatric adverse effects in glucocorticoid use is considerable, although causality cannot be inferred from noncomparative studies and potential underlying mechanisms are complex. Despite the need for more structured studies, it is important to acknowledge the potential of glucocorticoids to impact the brain and induce neuropsychiatric adverse effects given its use by a substantial proportion of the population. Upon starting glucocorticoid treatment, awareness of possible psychiatric side effects is essential.

## Data Availability

Original data generated and analyzed during this study are included in this published article or in the data repositories listed in References.

## Abbreviations

GR: glucocorticoid receptor
MR: mineralocorticoid receptor
RCT: randomized controlled trial
RoB 2: revised Cochrane risk of bias tool
ROBINS-I: risk of bias in non-randomized studies of interventions
SMD: standardized mean difference
